# First confirmatory study on *PTPRQ* as an autosomal dominant non-syndromic hearing loss gene

**DOI:** 10.1186/s12967-019-2099-5

**Published:** 2019-10-26

**Authors:** Dominika Oziębło, Anna Sarosiak, Marcin L. Leja, Birgit S. Budde, Grażyna Tacikowska, Nataliya Di Donato, Hanno J. Bolz, Peter Nürnberg, Henryk Skarżyński, Monika Ołdak

**Affiliations:** 1 Department of Genetics, World Hearing Center, Institute of Physiology and Pathology of Hearing, M. Mochnackiego 10, 02-042 Warsaw, Poland; 2 Postgraduate School of Molecular Medicine, Medical University of Warsaw, Warsaw, Poland; 3 Cologne Center for Genomics (CCG), University of Cologne, Cologne, Germany; 4 Department of Otoneurology, Institute of Physiology and Pathology of Hearing, Warsaw, Poland; 5 Institute for Clinical Genetics, TU Dresden, Dresden, Germany; 6 Senckenberg Zentrum für Humangenetik, Frankfurt am Main, Germany; 7 Center for Molecular Medicine Cologne (CMMC), University Hospital Cologne, Cologne, Germany; 8 Department of Oto-Rhino-Laryngology Surgery Clinic, Institute of Physiology and Pathology of Hearing, Warsaw, Poland

**Keywords:** Hearing loss, *PTPRQ*, Pathogenic, Dominant, Next-generation sequencing, Linkage

## Abstract

**Background:**

Biallelic *PTPRQ* pathogenic variants have been previously reported as causative for autosomal recessive non-syndromic hearing loss. In 2018 the first heterozygous *PTPRQ* variant has been implicated in the development of autosomal dominant non-syndromic hearing loss (ADNSHL) in a German family. The study presented the only, so far known, *PTPRQ* pathogenic variant (c.6881G>A) in ADNSHL. It is located in the last *PTPRQ* coding exon and introduces a premature stop codon (p.Trp2294*).

**Methods:**

A five-generation Polish family with ADNSHL was recruited for the study (n = 14). Thorough audiological, neurotological and imaging studies were carried out to precisely define the phenotype. Genomic DNA was isolated from peripheral blood samples or buccal swabs of available family members. Clinical exome sequencing was conducted for the proband. Family segregation analysis of the identified variants was performed using Sanger sequencing. Single nucleotide polymorphism array on DNA samples from the Polish and the original German family was used for genome-wide linkage analysis.

**Results:**

Combining clinical exome sequencing and family segregation analysis, we have identified the same (NM_001145026.2:c.6881G>A, NP_001138498.1:p.Trp2294*) *PTPRQ* alteration in the Polish ADNSHL family. Using genome-wide linkage analysis, we found that the studied family and the original German family derive from a common ancestor. Deep phenotyping of the affected individuals showed that in contrast to the recessive form, the *PTPRQ*-related ADNSHL is not associated with vestibular dysfunction. In both families ADNSHL was progressive, affected mainly high frequencies and had a variable age of onset.

**Conclusion:**

Our data provide the first confirmation of *PTPRQ* involvement in ADNSHL. The finding strongly reinforces the inclusion of *PTPRQ* to the small set of genes leading to both autosomal recessive and dominant hearing loss.

## Background

Hearing loss (HL) is the most common disability of human senses. It is considered to have a monogenic origin in at least half of the individuals developing HL prior to speech acquisition. Similar data are not available for postlingual HL but it is noticeable that familial aggregation of later-onset, progressive HL often follows an autosomal dominant pattern of inheritance. Up to now, 46 genes involved in the development of autosomal dominant non-syndromic HL (ADNSHL) have been identified (https://hereditaryhearingloss.org; accessed 10/2019). New genes related to ADNSHL are still being discovered and many of the identified variants in known HL genes represent novel changes. This highlights a great genetic heterogeneity of ADNSHL [1, 2].

In 2018, Eisenberger et al. [3] proposed *PTPRQ* (MIM*603317) as a new candidate gene for ADNSHL. The conclusion was based on the identification of a novel heterozygous nonsense *PTPRQ* variant p.Trp2294* that cosegregated with progressive HL in a German family. *PTPRQ* encodes protein tyrosine phosphatase receptor type Q, which catalyzes the dephosphorylation of phosphatidylinositol phosphates and displays a low activity against phosphotyrosine [4, 5]. Expression of *PTPRQ* in the inner ear is confined to hair bundles in the cochlea and in the vestibule and distributed depending on the hair cell type or its location, primarily in the basal region of hair bundles. In the cochlea, high PTPRQ protein level is detected in its basal turn responding to high frequency sound [6]. The main PTPRQ isoform in the inner ear contains a long extracellular domain, a short hydrophobic transmembrane region and an intracellular domain with a single phosphatase catalytic site [7].

The p.Trp2294* variant is predicted to introduce a premature termination codon (PTC) shortening the PTPRQ protein at the C-terminus by the last six amino acid residues. However, *PTPRQ* expression at the mRNA level remains unaffected, indicating that the mutant transcript escapes nonsense-mediated decay and supporting the hypothesis that the truncated PTPRQ protein is produced [3]. Performing additional functional studies to confirm the mutant variant mechanism of action was hampered by the large size of the PTPRQ protein and small difference in size between the wild-type and mutant protein. Since the initial report no additional data have been provided to unequivocally confirm *PTPRQ* as an ADNSHL gene.

Our study provides novel family-based evidence, confirming the pathogenic role of *PTPRQ* in ADNSHL. Based on the results of clinical exome sequencing, we identified the same *PTPRQ* variant as Eisenberger et al. in a new five-generation Polish ADNSHL family and found that both families have a common ancestor. Our strong genetic and clinical data constitute an ultimate confirmation for the causative role of *PTPRQ* in ADNSHL.

## Materials and methods

### Study subjects

**Fig. 1:**
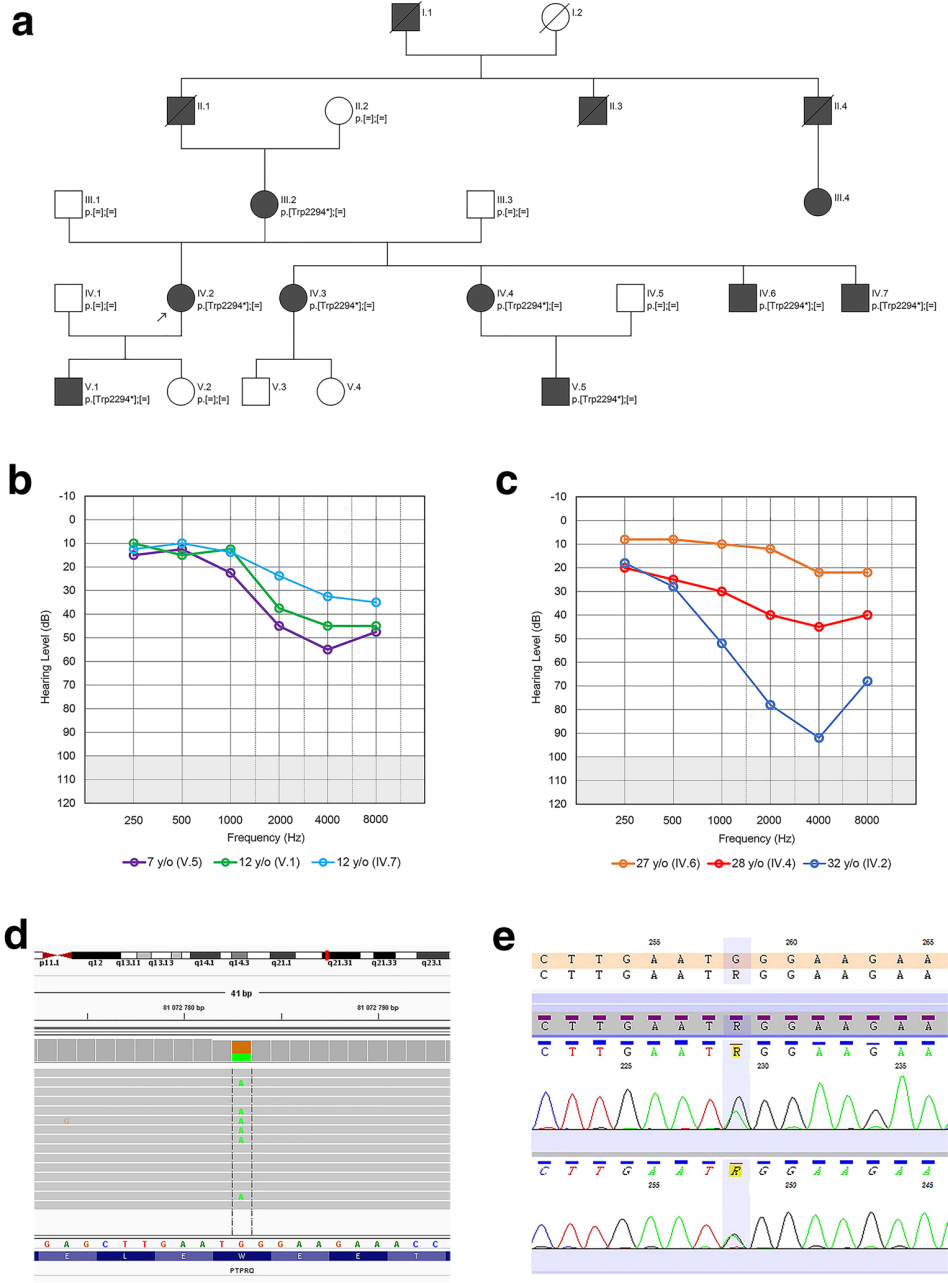
Identification of a heterozygous c.6881G>A *PTPRQ* pathogenic variant in a 5-generation ADNSHL family. **a** Family pedigree showing a typical autosomal dominant mode of inheritance and cosegregation of the *PTPRQ* variant with HL. The proband is marked with an arrow. Affected individuals are indicated by black symbols, unaffected individuals are indicated by open symbols, diagonal line denotes deceased family members. **b**, **c** Pure tone audiometry of selected family members at a similar age between 7 and 12 y/o (**b**) and 27–32 y/o (**c**) showing varying degrees of hearing loss. “O” symbols denote a mean binaural values of air conduction thresholds. **d** Clinical exome sequencing results visualized with the Integrative Genomic Viewer software illustrating the presence of a heterozygous guanine to adenine transition (c.6881G>A) (green letters) localized within exon 45. of the *PTPRQ* gene and resulting in a premature stop codon (p.Trp2294*). **e** The corresponding electropherogram from Sanger sequencing

A five-generation Polish family with progressive, high-frequency ADNSHL was recruited for the study (Fig. 1a). For linkage analysis, four family members of the previously reported German family were also included [3].

### Clinical evaluation

Auditory function of all participating family members was evaluated by pure tone audiometry. In the proband (IV.2) and her son (V.1) the examination was extended by impedance audiometry, transient evoked and distortion product otoacoustic emissions (TEOAE, DPOAE) and auditory brainstem responses (ABRs). Function of the vestibular system was assessed by cervical and ocular vestibular evoked myogenic potentials (cVEMP, oVEMP) with a 500-Hz tone burst at 97 dBnHL and Fitzgerald–Hallpike bithermal caloric stimulation with videonystagmography [8]. Anatomy of the auditory system was visualized by temporal bone computed tomography (Siemens CT Definition AS, Germany).

### Next-generation sequencing of clinical exome and Sanger sequencing

Genomic DNA of the 14 family members (8 affected and 6 unaffected) was isolated from blood samples with a standard procedure or from buccal swabs using Maxwell FSC DNA IQ Casework Kit (Promega, Madison, Wisconsin, USA) according to the manufacturer’s protocol. Next-generation sequencing of clinical exome was performed on proband’s DNA using TruSight One Sequencing Kit (Illumina, San Diego, California, USA) and a MiSeq platform (Illumina) with 2 × 150 bp paired-end reads according to the manufacturer’s protocol. All bioinformatic analyses were done as described previously [8]. Variants potentially related to ADNSHL in the family were selected based on the prediction scores from computational algorithms (including CADD, LRT, FATHHM, MutationTaster, PolyPhen-2, SIFT) and their frequency in population databases (including gnomAD, ESP6500, UK10K). Sanger sequencing using BigDye Terminator cycle sequencing kit v.3.1 (Applied Biosystems, Foster City, CA, USA) and 3500xL Genetic Analyzer (Applied Biosystems) was performed to confirm the presence and segregation of the identified *PTPRQ* and *TMC1* (MIM*606706) variants in all studied family members.

### Genome-wide linkage analysis

Genome-wide linkage analysis with DNA samples of the Polish (IV.1, IV.2, V.1, V.2) and German (DEIII.1, DEIII.2, DEIV.2 and DEIV.4 according to [3]) families was carried out using the Axiom Precision Medicine Research Array (Thermo Fisher Scientific, Waltham, MA, USA). Genotypes were called by the Axiom Analysis Suite v4.0 and processed as previously reported [3]. Multipoint LOD scores were calculated and haplotypes reconstructed with MERLIN [9].

## Results

Affected family members from the examined Polish family suffered from bilateral, progressive, high-frequency HL with an onset from 4 to 27 years of age. The degree of HL showed a high intrafamilial variability ranging from an almost normal hearing status to severe HL (Table 1). Detailed audiological evaluation revealed severe HL in the proband (IV.2, 32 y/o) and mild to moderate HL in her son (V.1, 12 y/o) at mid and high frequencies that was accompanied by tinnitus. At low frequencies hearing thresholds were within the normal range (Fig. 1a–c). In both individuals, tympanograms were normal and stapedial reflexes were present with ipsi- and contralateral stimulation with the exception of high frequencies where their thresholds were increased or absent. Otoacoustic emissions were bilaterally absent in the proband and recorded in her son up to 1500 Hz in the right and up to 1000 Hz in the left ear. Corresponding with the HL degree, ABRs recordings were lacking waves I and III, while wave V latencies were normal. Combined results of the objective hearing measurements showed bilateral hearing impairment originating from a defective cochlear component of the auditory system. Neurotological examinations showed cVEMP and oVEMP responses and results of bi-thermal caloric irrigation within normal limits. There were no malformations of the auditory system on temporal bone imaging (Additional file 1: Figures S1–S7).

**Table 1:** Clinical and genetic data of all affected family members

Pat. ID	Sex	Age (years)	*PTPRQ* genotype		Age of HL onset (years)	Age at PTA testing (years)	PTA results—hearing thresholds (dB)							
			cDNA level	Protein level			Tested ear	0.125 kHz	0.25 kHz	0.5 kHz	1 kHz	2 kHz	4 kHz	8 kHz
III.2	F	56	c.[6881G>A];[=]	p.[(Trp2294*)];[(=)]	8	54	R	N/A	25	25	20	45	50	70
							L	N/A	25	25	20	35	65	90
IV.2	F	36	c.[6881G>A];[=]	p.[(Trp2294*)];[(=)]	7	35	R	15	20	30	65	75	90	70
							L	15	20	30	60	65	80	60
IV.3	F	29	c.[6881G>A];[=]	p.[(Trp2294*)];[(=)]	28	28	R	N/A	20	15	15	40	40	60
							L	N/A	15	10	20	40	35	40
IV.4	F	32	c.[6881G>A];[=]	p.[(Trp2294*)];[(=)]	8	28	R	20	20	30	40	50	50	50
							L	30	20	20	20	30	40	30
IV.6	M	28	c.[6881G>A];[=]	p.[(Trp2294*)];[(=)]	27	27	R	N/A	10	5	10	20	25	20
							L	N/A	5	10	10	5	20	25
IV.7	M	21	c.[6881G>A];[=]	p.[(Trp2294*)];[(=)]	10	16	R	30	30	25	20	35	35	35
							L	30	30	25	25	35	35	45
V.1	M	14	c.[6881G>A];[=]	p.[(Trp2294*)];[(=)]	10	12	R	15	10	15	10	30	45	35
							L	15	10	15	15	45	45	55
V.5	M	9	c.[6881G>A];[=]	p.[(Trp2294*)];[(=)]	4	7	R	20	15	15	25	55	60	50
							L	15	15	10	20	35	50	45

Genotypes description using NM_001145026.2 and NP_001138498.1 reference sequences

*HL* hearing loss, *PTA* pure tone audiometry, *R* right, *L* left, *dB* decibels, *kHz* kilohertz, *F* female, *M* male, *N/A* not available, *=* normal allele

Molecular genetic testing showed the presence of a heterozygous NM_001145026.2:c.6881G>A variant in the *PTPRQ* gene, which fully segregated with ADNSHL in the studied family (Fig. 1a, d, e). The identified variant is located in the last coding exon of *PTPRQ* and introduces a PTC (NP_001138498.1:p.Trp2294*). The *PTPRQ* variant has not been reported in population databases but was detected in a single previously published German ADNSHL family [3]. Based on European descent and the close geographic proximity, a common ancestor was assumed. An affected and an unaffected offspring together with their parents were genotyped on a genome-wide single nucleotide polymorphism (SNP) array. The region surrounding the shared *PTPRQ* variant was analyzed for linkage in each family. The subsequent reconstruction of haplotypes revealed that a SNP haplotype spanning 306 kb (from rs1358476 to rs7978990) is shared by both families (Additional file 2: Figure S8).

In addition to the *PTPRQ* variant identified in the proband, we have found six other genetic alterations in HL genes that had population allele frequency below 0.001 and were present in protein coding regions. Considering the mode of HL inheritance and literature data, we have selected the *TMC1* NM_138691.2:c.1705A>G variant for family segregation study and found that it was present in normal hearing individuals and absent in some affected family members [10]. Its presence did not correspond with a more severe HL phenotype within the family.

## Discussion

Our report is the first confirmatory study on the causative role of *PTPRQ* in ADNSHL development. Here, we have identified eight previously unreported individuals suffering from progressive, high-frequency ADNSHL caused by the c.6881G>A *PTPRQ* nonsense variant (p.Trp2294*), which represents the first and so far the only *PTPRQ* pathogenic variant involved in ADNSHL. Up to now, it was reported exclusively by Eisenberger et al. [3] who, after detailed examination of a German family, proposed *PTPRQ* as a novel ADNSHL candidate gene (assigned at the DFNA73 locus 12q21.31). Their assumption was based on the results of exome sequencing, supported by linkage analysis, extended sequencing of the alternatively spliced exons and in silico predicted, potential *PTPRQ* coding regions.

In their paper, Eisenberger et al. have listed variants in genes reportedly associated with hearing impairment and variants located in the mapped candidate region encompassing *PTPRQ* that were included in the final analysis of potential ADNSHL pathogenic variants in the studied family. We have carefully compared the high throughput sequencing data of the German proband with the sequencing results of our proband and found that the majority of variants were not present in our patient except for variants within *SYT1* and *TMTC2* genes flanking *PTPRQ* on chromosome 12. Current data from the gnomAD database showed that both variants have a high population frequency (0.0023 and 0.0097, respectively) and are present in homozygous state in 2 and 43 individuals, respectively. This allowed to unequivocally exclude both genes from further consideration.

To verify whether the detected *PTPRQ* variant is located within the same inherited chromosomal region or represents an independent event that occurred by chance at the same position in both families, genome-wide linkage analysis was performed. After testing four family members from each family, we found that the *PTPRQ* c.6881G>A variant is present within a haplotype shared by the affected individuals from both families. It is located almost in the middle of a common chromosomal region containing the distal half of the *PTPRQ* gene together with the following 3′ region. The data have clearly demonstrated that the c.6881G>A containing *PTPRQ* chromosomal region did not undergo recombination and HL patients in both families share copies of the same part of the ancestral *PTPRQ* haplotype.

Here, for the first time we have performed deep phenotyping of the affected individuals and found that the defect caused by the pathogenic *PTPRQ* p.Trp2294* variant is restricted to the cochlear component of the auditory system. In contrast to individuals with *PTPRQ* recessive variants who suffer from vestibular dysfunction diagnosed in infancy or early childhood, our patients do not develop a vestibular disorder [11, 12].

The exact function of the PTPRQ protein remains poorly understood. Its large extracellular part forms shaft connectors most probably involved in proper spacing of stereocilia and maintaining their stability. The intracellular fragment containing an active enzymatic site was found to regulate the level of phosphatidylinositol phosphates. It has been also proposed that the PTPRQ C-terminus interacts with a protein complex containing MYO6, RDX, TPRN and CLIC5 [13], encoded by known HL genes. Proper function of the protein complex is required to maintain typical localization of PTPRQ at the base of stereocilia. In MYO6- and CLIC5-deficient mice, PTPRQ cannot be properly compartmentalized and it dissociates to the upper parts of the shafts leading to destabilization of membrane-cytoskeletal attachments, membrane lifting and consequent fusing or loosing of stereocilia [13–16].

The p.Trp2294* variant terminates the intracellular region of the PTPRQ protein after 30 amino acid residues following its catalytic site. It is the only *PTPRQ* pathogenic variant reported so far that results in a shortened PTPRQ protein with a preserved catalytic domain. One may speculate that the truncated PTPRQ may not be able to properly interact with its molecular partners and may diffuse and disturb the gradient of phosphatidylinositol phosphates along the shafts in a gain of function mechanism. Another possibility is that the pathologically shortened protein could disturb the function of the normal PTPRQ copy. It may exert a dominant negative effect and compete with the normal PTPRQ protein for binding of its molecular partners or co-participate in the multi-protein complex formation. As a consequence, the defective PTPRQ protein would weaken the protein complex and lead to insufficient stereocilia resistance to mechanical forces. This could modulate HL age of onset and severity which could explain the large intra- and interfamilial difference in this regard. In both studied families ADNSHL begins and progresses mainly at high frequencies adopting gradually a “ski-slope” curve on an audiogram, which is typical for HL induced by environmental factors.

## Conclusions

In summary, *PTPRQ* is better known as a recessive HL gene [11, 12, 17–21]. The present study strongly reinforces its inclusion to the small set of genes leading to both autosomal recessive and dominant hearing loss. Our data provide the first independent confirmation of *PTPRQ* causative role in ADNSHL.

## Supplementary information

**Additional file 1: Figure S1–S7.** Results of proband’s audiological evaluation.

**Additional file 2: Figure S8.** Linkage analysis for chromosome 12 in individuals from German (DEIV.2, DEIV.4) and Polish (V.1, V.2) families.

## Abbreviations

ABRs: auditory brainstem responses
ADNSHL: autosomal dominant non-syndromic hearing loss
cVEMP: cervical vestibular evoked myogenic potentials
DPOAE: distortion product otoacoustic emissions
HL: hearing loss
oVEMP: ocular vestibular evoked myogenic potentials
PTC: premature termination codon
SNP: single nucleotide polymorphism
TEOAE: transient evoked otoacoustic emissions

## Availability of data and materials

Please contact author for data requests.
