# Supplementary material for: First confirmatory study on PTPRQ as an autosomal dominant non-syndromic hearing loss gene
Source: J Transl Med. 2019 Oct 26;17:351. doi: 10.1186/s12967-019-2099-5 (PMC6815010; doi:10.1186/s12967-019-2099-5)
Supplement: Supplementary file 1 — Additional file 1: Figure S1–S7. Results of proband’s audiological evaluation. [file 12967_2019_2099_MOESM1_ESM.pdf]

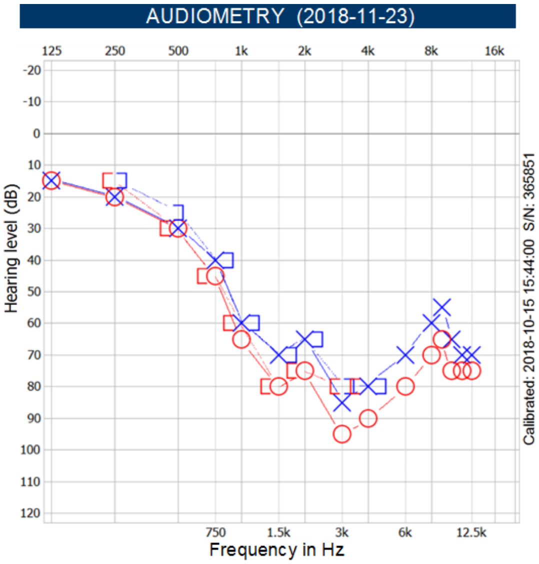

Figure S1 Pure tone audiometry results of proband (IV.2, 35 y.o.). Red "O" and blue "X" symbols denote air conduction thresholds in the right and left ear, respectively and "[" or "]" denote masked bone conduction. Down-sloping sensorineural hearing loss is observed.

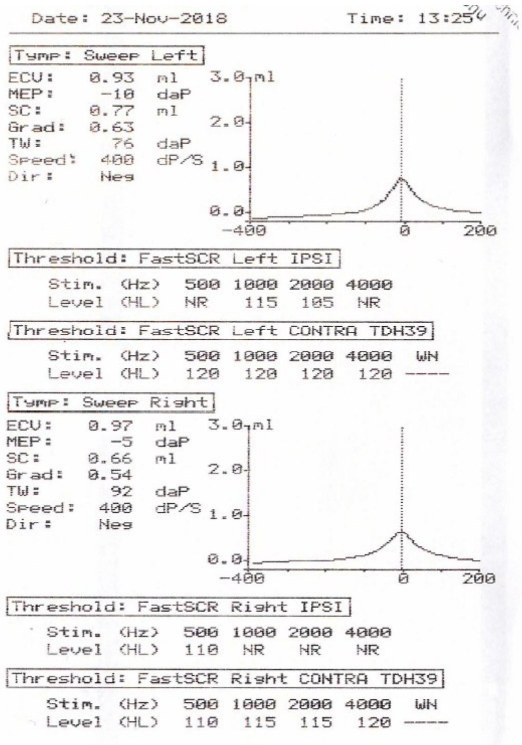

Figure S2 Tympanometry and acoustic reflex measurements in proband (IV.2, 35 y.o.). Tympanograms bilaterally within normal limits, acoustic reflex present for ipsi- and contralateral stimulation. For frequencies with a high degree of HL, acoustic reflex tresholds are elevated or absent for ipsilateral stimulation.

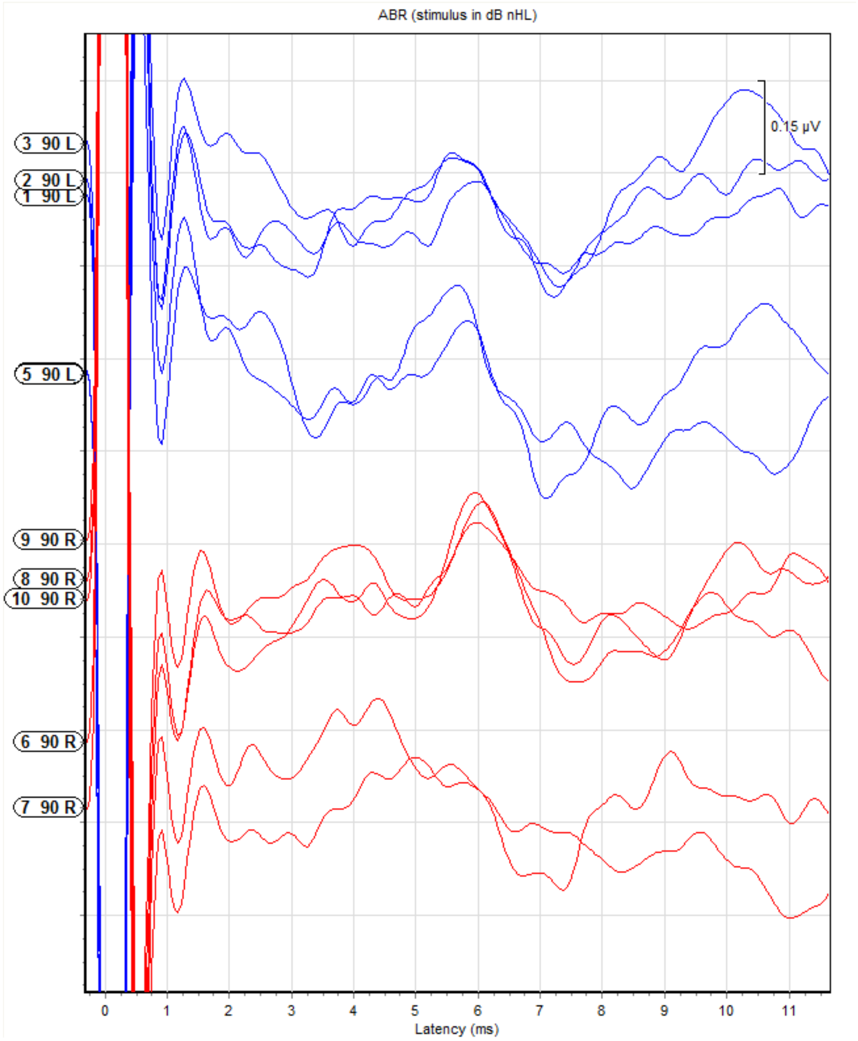

Figure S3 Auditory brainstem responses recordings in proband (IV.2, 35 y.o.). Measurements after click stimulus at an acoustic level of 90 dB normal hearing level (nHL) and presentation rate of 11/s. Bilaterally absent waves I-III which corresponds with the degree of HL. Wave V latencies within normal limits.

A

Right ear

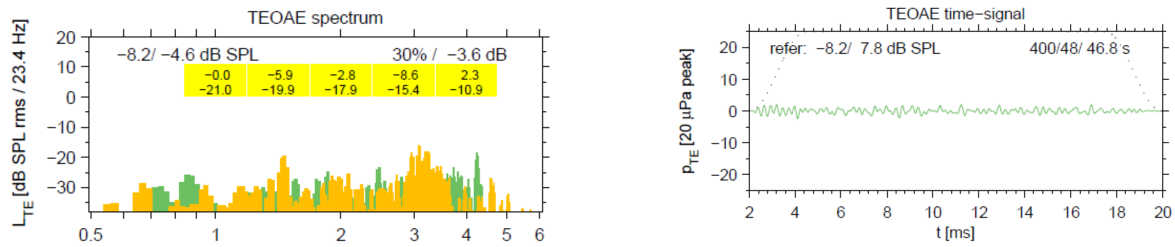

Left ear

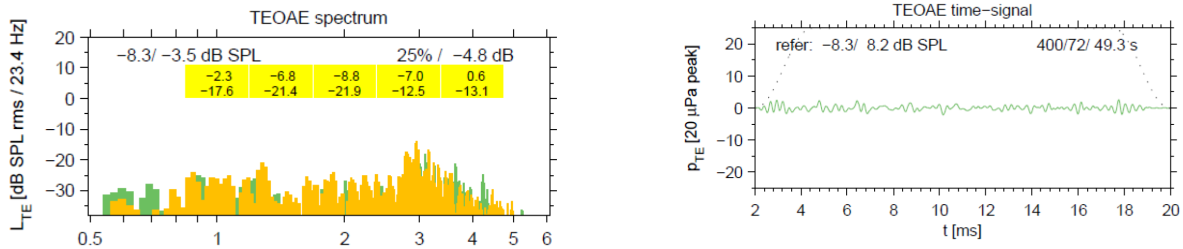

B

Right ear

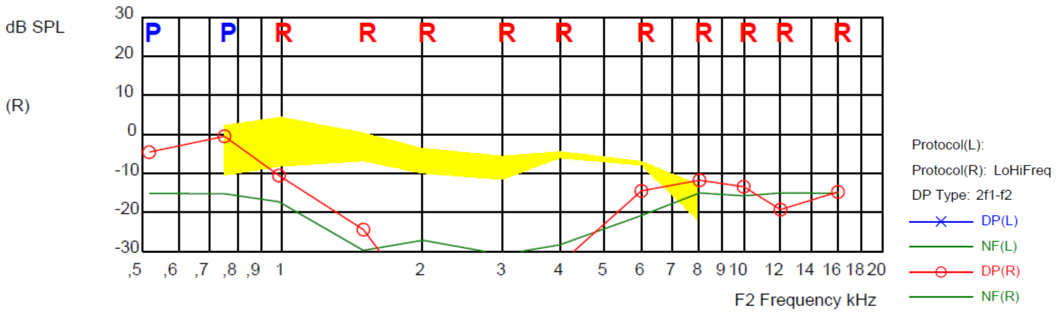

Left ear

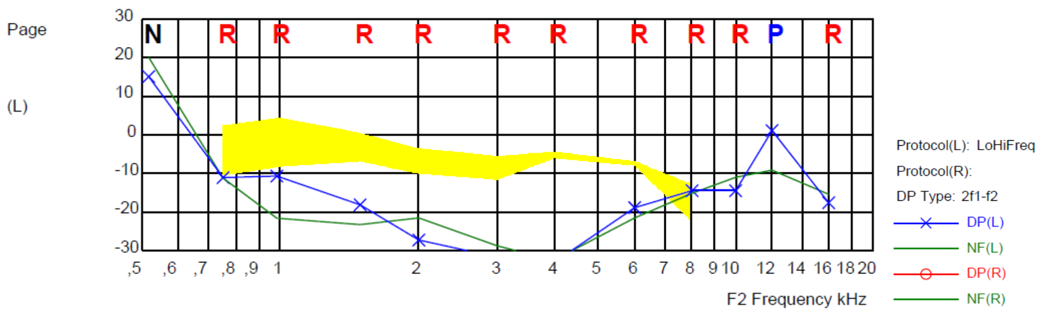

Figure S4 Transient evoked (A) and distortion products (B) otoacustic emissions measurements in proband (IV.2, 35 y.o.). Bilaterally otoacoustic emissions are absent.

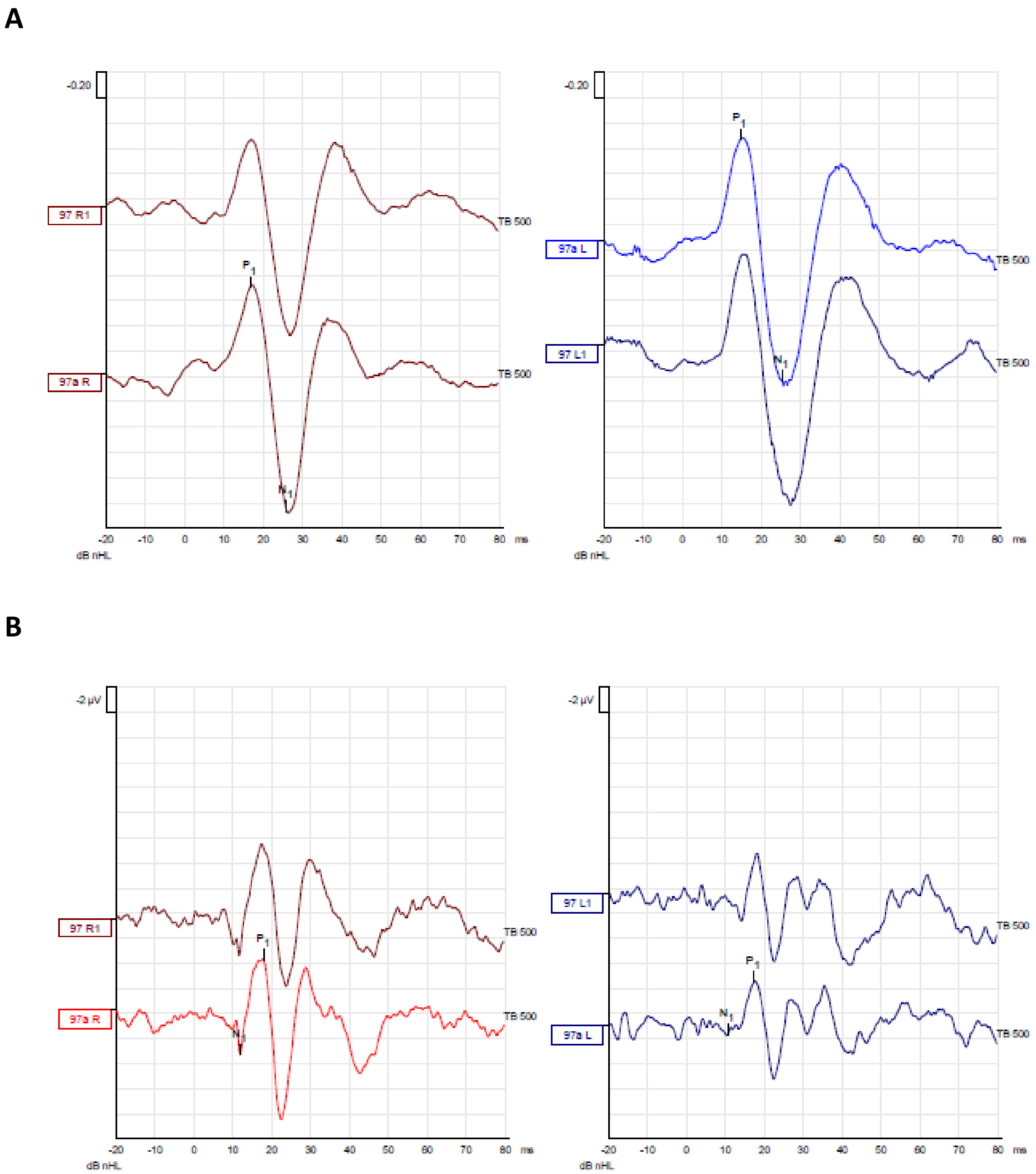

Figure S5 Cervical (A) and ocular (B) vestibular evoked myogenic potentials (VEMP) recordings in proband (IV.2, 35 y.o.). VEMPs are bilaterally within normal limits.

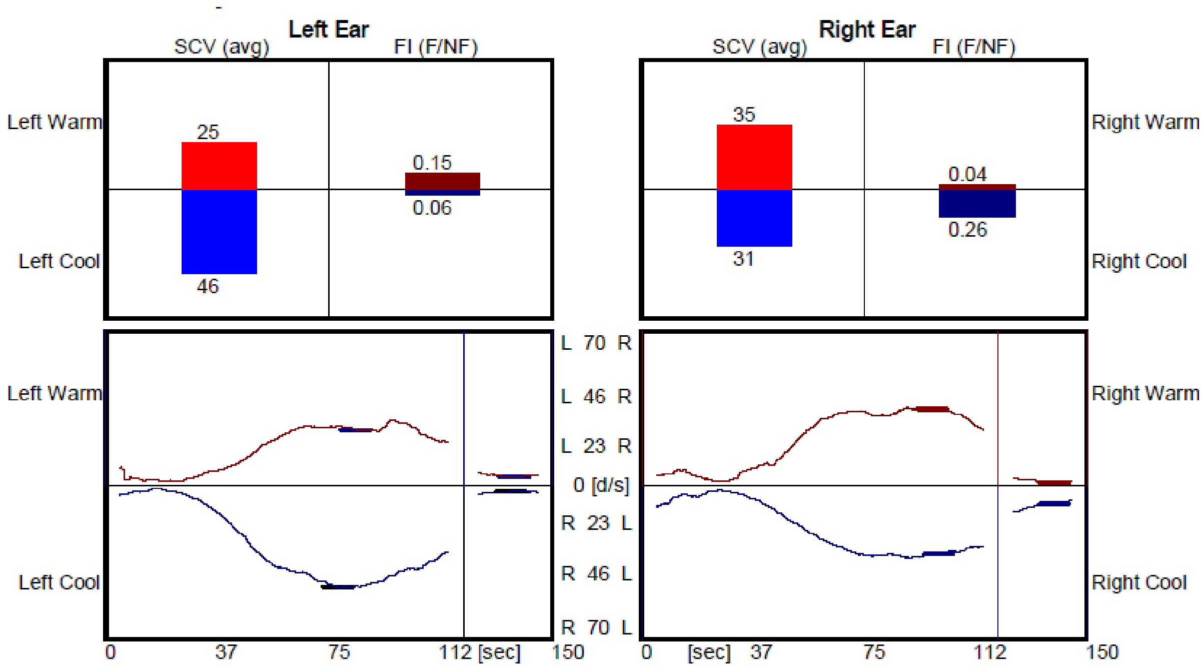

Figure S6 Caloric test results in proband (IV.2, 35 y.o.). A caloric Reduced Vestibular Response (RVR) is within normal limits.

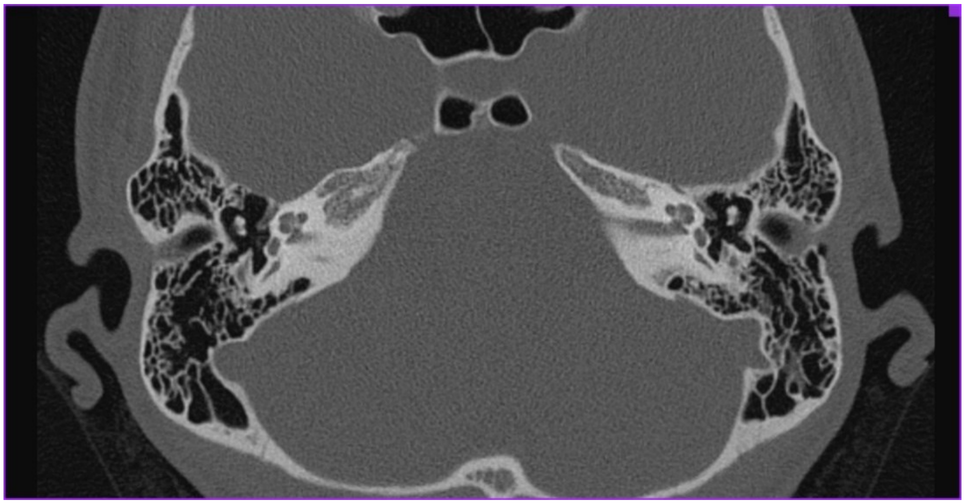

Figure S7 Computed tomography of temporal bones imaging in proband (IV.2, 35 y.o.). The inner ear structures and ossicles are normal.
